# A decade in review: use of data analytics within the biopharmaceutical sector

**DOI:** 10.1016/j.coche.2021.100758

**Published:** 2021-12

**Authors:** Matthew Banner, Haneen Alosert, Christopher Spencer, Matthew Cheeks, Suzanne S Farid, Michael Thomas, Stephen Goldrick

**Affiliations:** 1 Department of Biochemical Engineering, University College London, Gower Street, London WC1E 6BT, UK; 2 Cell Culture Fermentation Sciences, Biopharmaceutical Development, BioPharmaceuticals R&D, AstraZeneca, Cambridge, UK; 3 London Centre for Nanotechnology, University College London, Gordon Street, London WC1H 0AH, UK

## Abstract

There are large amounts of data generated within the biopharmaceutical sector. Traditionally, data analysis methods labelled as multivariate data analysis have been the standard statistical technique applied to interrogate these complex data sets. However, more recently there has been a surge in the utilisation of a broader set of machine learning algorithms to further exploit these data. In this article, the adoption of data analysis techniques within the biopharmaceutical sector is evaluated through a review of journal articles and patents published within the last ten years. The papers objectives are to identify the most dominant algorithms applied across different applications areas within the biopharmaceutical sector and to explore whether there is a trend between the size of the data set and the algorithm adopted.

## Highlights

• Data analytics has increasing significantly in recent years in the biopharma sector.
• No clear trend observed between algorithm utilisation and data size.
• PLS was found to be most applied algorithm within the biopharmaceutical sector.
• Majority of the data analytics applications are focused on USP operations.
• Data analytics will play a key role as the sector transitions towards Industry 4.0.

**Current Opinion in Chemical Engineering** 2021, **34**:100758

This review comes from a themed issue on **Biotechnology and bioprocess engineering: mechanistic and data-driven modelling of bioprocesses**

Edited by **Cleo Kontoravdi** and **Colin Clarke**

For a complete overview see the Issue

Available online 15th November 2021

**https://doi.org/10.1016/j.coche.2021.100758**

2211-3398/© 2021 The Authors. Published by Elsevier Ltd. This is an open access article under the CC BY license (http://creativecommons.org/licenses/by/4.0/).

## Introduction

The biopharmaceutical sector has seen significant improvement in bioreactor design, instrumentation and analytical technologies over the last 10 years. One of the most widely adopted technological advancements within the sector is the utilisation of high-throughput automated micro-bioreactors, enabling the parallelisation of experiments using a fraction of the liquid volume required for laboratory-scale experiments [1]. Additionally, there has also been a large push from regulatory bodies to adopt process analytical technology (PAT) for improved monitoring and control of biopharmaceutical processes [2,3]. Recent advances include also the application of omics, such as transcriptomics, proteomics, metabolomics and fluxomics in the sector, allowing for a better understanding of the intracellular workings of cellular processes [4]. These recent advancements have resulted in the generation of larger and more diverse data sets requiring more specialised statistical and modelling tools for efficient data analysis. The process of data analytics can be described as the analysis of raw data to make useful conclusions about the information provided. The application of these tools enables the true potential of data to be fully harnessed for improved process understanding and more informed decision-making [5] facilitating optimisation of existing biomanufacturing processes and hence increased product quantity and quality. The increased use of these advanced sensors and automated bioreactors will play a key role in the advancement of the sector towards the core principles of Industry 4.0 [6]. However, to ensure these ‘smart factories’ of the future can deliver improved and automated process control, the need for advanced data analytics is paramount.

The process of data analytics is predominantly carried out using machine learning (ML) algorithms. ML is a very broad term which can be defined as the generation of algorithms to perform tasks based on rules learnt from the data rather than explicitly programmed by the user. The application of these algorithms within the biopharmaceutical sector can provide additional insights and identify patterns within data sets enabling improved decision making for process optimisation. Certain ML algorithms such as neural networks (NN) and random forests (RF) are typically considered computationally intensive which has previously hindered their widespread application, however there has been a recent surge in the application of these algorithms due to the increase in computing power available and improvements in algorithm efficiency [7]. ML algorithms have seen also an increase in popularity and impact across other sectors outside the biopharmaceutical sector, such as analysing markets to predict better performing stocks in the finance sector [8] and targeted display adverts in the advertising sector [9]. Traditionally within the biopharmaceutical sector, a subset of ML referred to as multivariate data analysis (MVDA) has been used to examine variable interactions and is preferred over univariate and bivariate techniques due to its ability to analyse multiple variables and minimise false inferences [10,11]. MVDA algorithms such as partial least squares (PLS) and linear regression (LR) are commonly used in the sector. Examples include the application of a PLS algorithm for the prediction of amino acid concentrations by analysing Raman spectroscopy in mammalian cell cultures [12] and the application of LR for root cause analysis of product quality deviations in therapeutics proteins [13].

With the growing body of ML algorithms, it can be a challenge to decide which algorithm to implement. Within this study, an evaluation of data analysis techniques was carried out over the period of 2010–2020 to identify any trends related to the utilisation of specific algorithms within bioprocessing. Initially, the paper reviews the rising prevalence of data analysis techniques in the sector by evaluating literature and patents published between the period of 2010–2020. A more in-depth analysis of the most cited literature from the period of 2015–2020 was carried out to identify the most dominant algorithms, the size of the data sets utilised within each model and their application area within bioprocessing. This paper outlines a clear increase in the application of data analytics within the biopharmaceutical sector and demonstrates the wide range of algorithms implemented to analyse these complicated bioprocessing data sets. The increased use of these digital methodologies demonstrates the shift of the sector towards Industry 4.0, which envisages fully automated and autonomous biomanufacturing operations.

## Material and methods

The methodology required to produce this analysis is split into two parts: a systematic search of all scientific literature containing the key phrases: MVDA, ML and biopharmaceuticals, assessed between the period of 2010–2020 and an in-depth analysis of the most impactful journal articles assessed from the period of 2015–2020.

### Literature search (2010–2020)

Google Scholar was queried to search scientific literature published from 1 January 2010 to 31 December 2020 that mentioned the key terms ML or MVDA and biopharmaceutical. The exact search terms for each query were:

• MMVDA - (“Bioprocess” OR “Biopharmaceutical”) AND (“Multivariate data analysis” OR “Multivariate analysis”)
• ML - (“bioprocess” OR “biopharmaceutical”) AND (“machine learning” OR “artificial Intelligence”)

Similarly, for patents, Google Patent was queried using the aforementioned search terms to gather patents that contained the key terms MVDA or ML and biopharmaceutical between 1 January 2010 and 31 December 2020. For both patents and journal articles, the total number of results were recorded for each year.

To evaluate the overall relevance factor, the number of patents and journal articles for both MVDA and ML were quantified using the formula defined as the ‘Relevance Index’, $\;R_{i}$:

(1) $$R_{i}=\;{0.5\;(J}_{Si})+\;0.5\;(P_{Si}\text{)}\text{ for}\text{ i = 2010,……,2020}$$

where $J_{Si}$ is the standardised number of journal article results of the ith year and $P_{Si}$ is the standardised number of patent results of the ith year.

Where the standardised values are defined as:

• Journal articles
  (2) $$J_{Si}=\;J_{i}/\text{m}\text{a}\text{x}\;(J)\text{ for}\text{ i = 2010,……,2020}$$
  where $J_{i}$ is the number of journal article results at the ith year.
• Patents
  (3) $$P_{Si}=\;P_{i}/\text{m}\text{a}\text{x}\;(P)\text{ for}\text{ i = 2010,……,2020}$$
  where $P_{i}$ is the number of patent results at the ith year.

### Literature analysis (2015–2020)

To assess the most impactful articles published over the last five years, the key journal articles were defined as those that had the highest average citation number per year since publication. Whilst the use of ‘citation number’ to evaluate the impact of a journal article has its limitations, they are considered to be a good measure of scientific impact and relevance [14]. For the purpose of this paper, this metric enables potential trends to be identified in the biopharmaceutical sector. The key journal articles were identified using the software Harzing’s Publish or Perish [15]. Harzing’s Publish or Perish scrapes the search results from Google Scholar using the same search terms mentioned previously and tabulates and cumulates the data. For each year between 2015 and 2020, the top 10 most cited journal articles for each year about data analytics were collected. From these 60 journal articles, the metadata was manually extracted. This included:

• the algorithm used,
• the number of experiments used in the analysis,
• the number of variables used in the analysis,
• the application area in terms of problem domain (e.g. fault detection) and process stage (e.g. upstream processing (USP), downstream processing (DSP) or other manufacturing areas).

The data collected only considered the top 60 journal articles which defined the number of variables and number of experiments analysed within their study. Where journal articles evaluated multiple algorithms for their data analysis, the best performing algorithm was selected for this paper.

### Data analysis and visualisation

The metadata was imported, analysed and visualised using R 4.0.2 and Python 3.8.3.

## Results and discussion

### Prevalence of data analytics adoption in the biopharmaceutical sector

**Figure 1:**
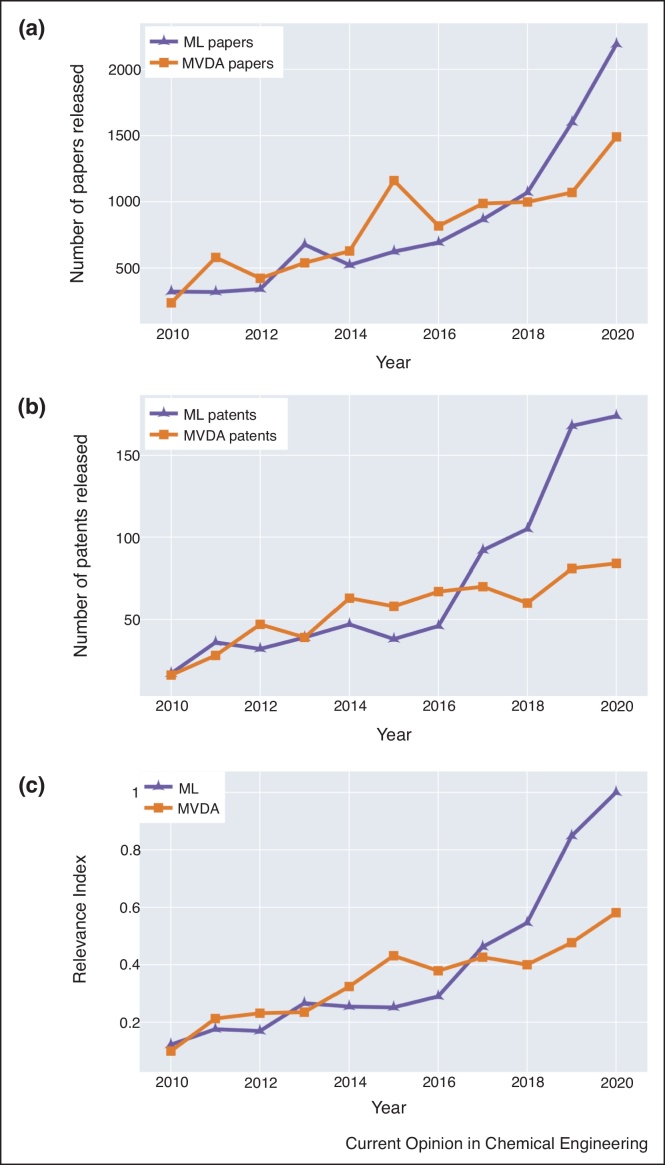
Prevalence of ML and MVDA data analytics adoption in the bioprocessing and biopharmaceutical sector during the period of 2010–2020 in terms of **(a)** journal articles, **(b)** patents and **(c)** the relevance index. The specific search criteria are provided in the Materials and Methods. The orange squares represent the search terms related to MVDA, and purple triangles represent those related to ML.

As the industry undergoes a digital transformation with the scale and complexity of the data sets increasing, there is a need to better understand how data analytics is used within the biopharmaceutical sector. To quantify the prevalence of MVDA and ML, a literature and patent search was carried out across the period of 2010–2020 using the search terms defined in the material and methods section with the aim of identifying potential trends in the utilisation of these data analysis techniques. The distinction in search terms was needed as some algorithms are exclusively referred to as MVDA while others are labelled exclusively as ML, therefore these search terms provide broader understanding of the utilisation of data analytics across the sector. Figure 1 compares the results recorded for MVDA and ML in terms of number of patents and journal articles released over the period of 2010–2020. To simplify the interpretation of these two metrics, a standardised plot of Relevance Index (Eq. (1)) is presented consolidating both the impact of journal articles and patents (Figure 1c). This metric enables a single evaluation of these search terms and helps determine their utilisation in the sector more broadly.

Figure 1a–c show a positive trend demonstrating that ML and MVDA are becoming more utilised in the biopharmaceutical sector. The utilisation of ML within the last five years has sharply increased by 250% for journal articles and 357% for patents. However, MVDA has only increased by 28% for journal articles and 44% for patents in the same period. The rise in the application of these techniques is most likely due to an increase in the complexity, size and format (e.g. images) of data available for analysis. As a result, more advanced algorithms are needed to analyse these complex data sets. Additionally, due to the recent advances in computational power and access to high-performance machines, the ability to train and validate ML algorithms on large complex data sets is now much easier. It is worth noting that some techniques that have historically been referred to as MVDA, such as PCA and PLS, are now often labelled as ML due to the increasing popularity of the latter term. The increase in published patents related to data analytics shown in Figure 1b may suggest that ML and MVDA are now becoming more adopted by industry. A recent patent by De Kok *et al.* demonstrated the value of implementing various non-linear ML techniques such as RFs and k–nearest neighbour to predict the performance of large scale systems through experimental design of small scale systems [16]. Furthermore, a recent patent utilising a PLS algorithm was published by Berry and Moretto for the analysis of Raman spectra to predict a number of culture parameters including glucose, lactate and ammonia for bioreactors ranging from 0.1 L to 100 000 L [17].

An additional consequence of the recent advances in computational power is the increased availability of commercial software that lowers the knowledge barrier to implement ML models. However, this can potentially lead to incorrect applications without full understanding of the detailed assumptions, considerations, and model limitations. For example, models must be able to account for large quantities of noise and variability that is common in biological systems due to their stochastic nature [18]. Another important consideration for model building is the interpretability of the model being utilised. Some ML algorithms, such as NN, are typically much more difficult to interpret due to their complex structures. This is particularly important in drug discovery where the selection of new treatments without justification may hinder regulatory approval. In these cases, further analysis and evaluation of a model’s performance and robustness is needed before implementation [19]. If these conditions are not considered, the risk of inaccurate predictions increases resulting in possible process failure and hence, financial loss.

To improve the adoption of ML algorithms within the biopharmaceutical sector, organisations need to ensure the correct architecture of their data storage facilities. Full and easy access to all available data within a structured and queryable database such as a data lake or warehouse will significantly simplify the application of ML algorithms. Some of the more complex ML algorithms often require larger data sets and more computational power is required to build and train these models. Therefore, a company’s data infrastructure becomes a priority.

Overall, ML has become a widely adopted technique within the biopharmaceutical sector based upon our analysis of journal articles and patents used in the sector between the period 2010–2020. The increased utilisation of both MVDA and ML algorithms within the biopharmaceutical industry is likely to continue. It is most likely to accelerate as the sector further adopts these algorithms for better decision-making within clinical and commercial manufacturing, however, there is little guidance in the sector as to how much data is needed for each technique or which algorithm will perform better. There are numerous examples where data analytics techniques are directly compared. An extensive analysis by Mendez *et al.* compared multiple non-linear ML and MVDA algorithms to classify ten clinical metabolomics data sets. They concluded that there was no general improvement in predictability between the non-linear and linear algorithms utilised. It was reiterated that ‘*a model is only as good as the data that is used to train it’* suggesting that the data set used to train models is an equally important factor as the algorithm performing the analysis [20^•^]. As such, an analysis on data size and algorithms applications is needed to identify potential trends.

### Classification and application of ML in the biopharmaceutical sector based on recent literature

**Figure 2:**
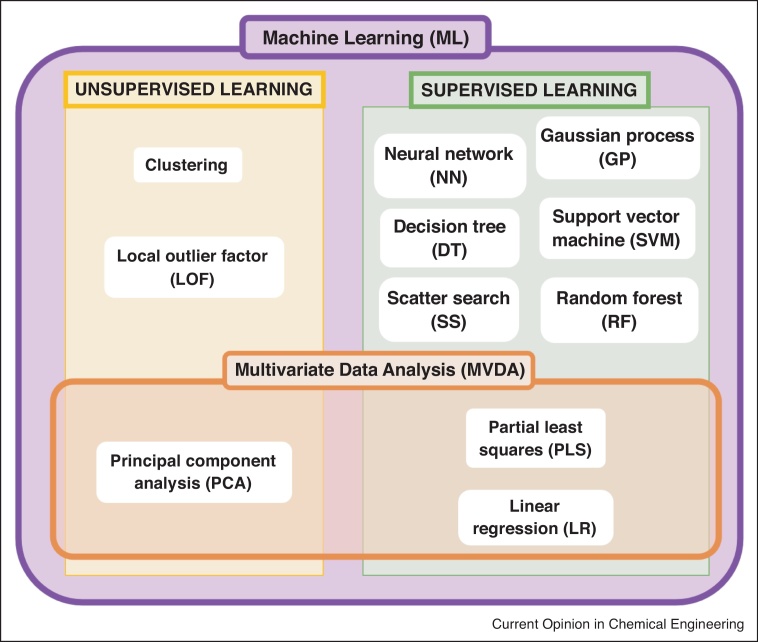
Classification of each algorithm employed in the top 60 journal articles based upon average citation number from the period of 2015–2020 identified as MVDA or ML and supervised or unsupervised learning technique. MVDA algorithms labelled as: Linear Regression (LR), Partial Least Squares (PLS) and Principal Component Analysis (PCA). ML algorithms labelled as: Clustering, Decision Tree (DT), Local Outlier Factor (LOF), Neural Networks (NN), Random Forests (RF), Support Vector Machines (SVM), Gaussian Process (GP) and Scatter Search (SS).

To better understand the most prominent application areas and the most dominant algorithms used within the biopharmaceutical sector over the period of 2015–2020, an in-depth evaluation of the most cited journal articles was conducted. The 10 most cited journal articles based upon average number of citations per year was recorded, which resulted in a total of 60 journal articles. Within each of these journal articles: the specific algorithms utilised, the size of the data sets analysed and whether the authors classified the algorithm as either MVDA or ML was documented. Patents were not considered in the in-depth analysis due to a lack of databases that could be queried for information. A summary of all the algorithms utilised within these 60 journal articles during this period is shown in Figure 2, with the learning method of each algorithm defined as either supervised or unsupervised. Supervised learning techniques require a labelled training set to build the model and establish relationships between the inputs and outputs of the given system [21]. Alternatively, unsupervised learning uses unlabelled data and focuses on identifying patterns within the data with the purpose of partitioning the data set into smaller subsets that have similar variable characteristics [21]. In total, there were 11 unique algorithms identified within the top 60 most cited journal articles using data analytics during the period of 2015–2020. As previously discussed within the biopharmaceutical sector, some of the traditional statistical algorithms such as PCA, PLS and LR have been labelled as MVDA within these journal articles. However, these algorithms are more broadly defined as a subset of ML although their exact definition can vary between disciplines.

There is a long history of utilising conventional MVDA algorithms such as PCA and PLS, which assume linear relationships between inputs and responses. PCA has also been extensively used to better understand root cause of batch-to-batch variations and has been successfully implemented as far back as 1987 [22] and is commonly used today [1]. PLS algorithms have been employed historically to monitor end-point quality of fermentations based on the golden batch concept. This allows for operators to identify the source of process deviations or disturbances from an ideal trajectory and take corrective action quickly [23]. More recently, they have proven useful for spectral analysis involving PAT applications [24^••^]. Other established ML algorithms include NN and support vector machines (SVM). These algorithms allow for non-linear relationships to be modelled, which can be particularly useful for capturing non-linearities within biological systems. For example, NN were applied successfully to predict loading capacity of depth filtration filters based upon non-linear functional correlations between inputs and outputs [25]. SVM are also advantageous due to their strong ability to generalise properties on unseen data which has resulted in its application across biological areas [26]. For example, SVM have been used to address the non-linear problem of predicting key process variables over time in penicillin production [27]. In-depth descriptions of the majority of the ML algorithms shown in Figure 2 can be found in the literature [28,29].

**Figure 3:**
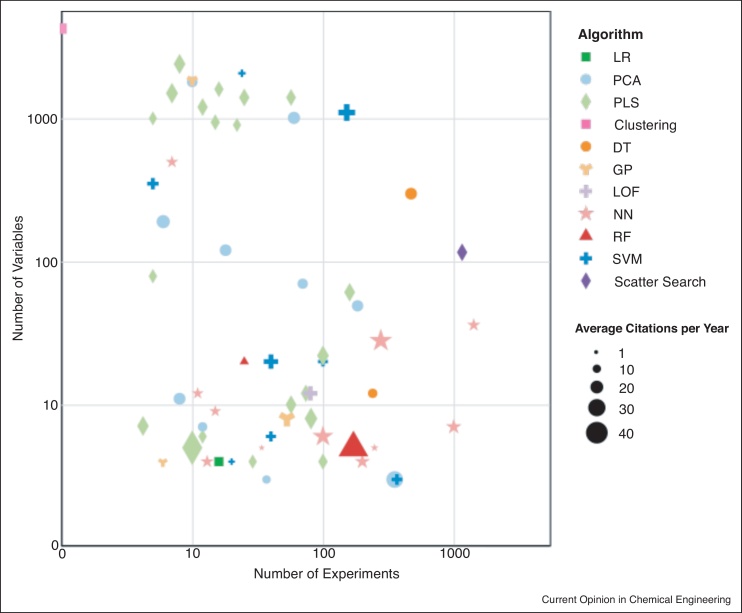
Characteristics of algorithms in the top 60 most cited data analytics journal articles from the time period of 2015–2020 in terms of number of variables and experiments (log scale), algorithm type and average citations per year. Each symbol represents a different algorithm. The size of the symbol is proportional to the average number of citations per year for the journal articles, where a bigger symbol indicates a higher number of citations.

Within each journal article, the information related to the size of the data sets analysed and the application area was extracted. The two major factors that were recorded from each journal article was the number of variables and the number of experiments within each of the data sets analysed by these algorithms. For this analysis, the definition of a variable is any information that was recorded throughout the experiment and an experiment was considered as a single independent process run. The extracted information regarding the number of experiments, number of variables, algorithm type and average citation per year of the top 60 data analytics journal articles is summarised in Figure 3.

The average number of variables within the data set analysed using data analysis techniques was 444 ± 801 (minimum 1) demonstrating that there is a large range in the number variables being analysed within each data set. As the number of variables increase, PLS becomes the most commonly used algorithm. This can be seen in Figure 3, where for 500 or more variables, approximately 60% of the journal articles utilised PLS. PLS is the one of the most commonly used algorithms for the analysis of spectral data sets, based on its proven ability to correlate large numbers of variables (i.e. spectral wavelengths) with either the critical quality attributes or critical process parameters of interest. The maximum number of variables shown in Figure 3 was taken from Lempp *et al.* who analysed 4242 transcripts related to different genes of *Escherichia coli* [30]. This data was recorded using a single 1 L bioreactor with high-frequency transcriptomics data measuring a total of 29 different time points across the 20-hours experiment. Within their analysis, they selected a hierarchical clustering algorithm and used this to classify how different gene pools affected the transcription concentrations during the bioreactor run. This demonstrates that successful models can be built using a single bioreactor run provided that high frequency analytics are implemented to ensure sufficient data is available to validate the model. However, the information captured on a singular run may not be generalised for the process as it will not account for batch-to-batch variations or differences in bioreactor operation. One of smallest numbers of variables shown by Figure 3 was taken from Villain *et al.* who used three variables to build an SVM model which modelled a quantitative structure activity relationship (QSAR) for the acute toxicities of algae [31]. To avoid overfitting, the data set of 368 experiments was used to cross validate the model using a threefold cross validation technique. While the number of variables is small, this demonstrates that models built from smaller number of variables can still have high predictive power. Typically, having more variables within a data set allows for more complex models to be built as there are more degrees of freedom within the data set allowing for more interactions and relationships between the variables to be defined. However, complex models are not always desirable as they can be difficult to interpret and also the risk of these models being overfitted becomes higher [32]; therefore, independent external validation data is advised to ensure the model is robust. The average number of experiments used for the analysis within each of these journal articles was equal to 128 ± 267 (minimum 1) with the largest number of experiments identified in Figure 3 from Riba *et al.* [33]. Riba *et al.* utilised 1423 images to train an NN to classify dispensed cells as viable or dead [33]. For the purpose of this analysis, each individual image was classified as an individual experiment. Each image consisted of a 50 by 50-pixel grid which was used to train and validate the model. To avoid overfitting, the prediction performance of the model was evaluated using a 10-fold cross validation procedure. The translation of images to information results in large quantities of data being produced which is well suited for algorithms such as NN as these algorithms are more data hungry and generally require more data to build accurate models [34]. However, it is observed in Figure 3 that NN have been implemented also to applications with much smaller data sets, which demonstrates the robustness of this algorithm to varying volumes of available data.

It was observed in Figure 3 that data analysis techniques have been successfully applied with experiment numbers as low as one. Another example of an application which utilised a small number of experiments to build a valid model was by Brestich *et al.*, who successfully applied PLS to UV-vis spectroscopy to monitor preparative chromatography [35]. This was achieved through the utilisation of three experiments to train the PLS model with one for validation, in addition to employing cross validation to avoid overfitting the model. While the number of experiments was small, the dimension of these data sets used was large. For each of the four different chromatography experiments, a spectrum from 240 nm to 300 nm with 2 nm resolution was obtained. As a result, the data set produced had large dimensionality, that is, a large number of variables, allowing for more complex models to be built that could yield accurate predictions. This is a common feature of the majority of other PATs in which large amounts of information can be recorded in a small number of experiments. Additional data analysis applications involving a smaller number of experiments has also been shown in Figure 3, however not all small data sets contain enough information to build valid models. Indeed, the study by Vodopivec *et al.* analysed a data set of five experiments and applied SVM to compare metabolic profiling of 350 different metabolites between bioreactors of different sizes. The authors concluded that the models produced were of low quality, potentially due to the lack of data regarding the various metabolites which were not considered during the bioreactor runs [36]. Furthermore, this indicates that the data sets used to train these models were not representative of the whole bioreactor system. Another journal article with a small number of experiments was by Wang *et al.* who applied Gaussian process (GP) multilinear regression to infer the modulation effect of four metabolites using six experiments as a training set and one for validation. A smaller number of experiments was sufficient as GP uses the generated estimates of modulation effects to estimate parametric models, which generate more data which resulted in more accurate predictions [37]. These applications demonstrate that smaller numbers of experiments can produce robust and accurate models, but the data needs to be representative of the whole process being modelled. It must be noted that for the analysis of any data set, there are numerous algorithms that can be applied and should yield similar prediction errors or find the same correlations. Therefore, the choice of algorithm is most likely highly dependent on the familiarity and experience of the user analysing the data. They will most likely apply their preferred algorithm first and if the results are satisfactory, this algorithm will be utilised in the model building process.

A common perception from the sector is that developing a robust model requires a large number of experiments. However, the majority of processes in the biopharmaceutical industry suffer from something referred to as the ‘Low-N’ problem, where there is a limited number of historic experiments available for modelling a particular process [38^•^]. The Low-N scenario is common particularly with new biopharmaceutical products which have often only one or two experiments or runs at manufacturing scale. In these cases, it is common that the number of experiments is less than the number of variables being considered. Building models using these data sets are at risk of being overfitted [39], particularly when the ratio of variables to experiments is large. Possible solutions to provide larger data sets necessary for some of these data-hungry models include using algorithms such as GP to generate artificial data based on small data sets [40]. The method mentioned by Tulsyan *et al.* assumes that the initial data sets being replicated are representative of the whole process, which may not be accurate. Other solutions involve the use of Digital Twins to generate unlimited simulated data sets. This data can be used to develop and evaluate ML algorithms for process optimisation and speed up the readiness of these algorithms to implement once experimental data becomes available [41]. Other challenges have been raised by Mowbrey *et al.* about the data produced in the biopharmaceutical sector. This includes the sparsity of high dimensional data sets that often do not contain sufficient information about each individual dimension (variable) for model building [29]. The authors proposed a solution to effectively utilise these data sets by filling in the knowledge gaps using first principle models.

**Figure 4:**
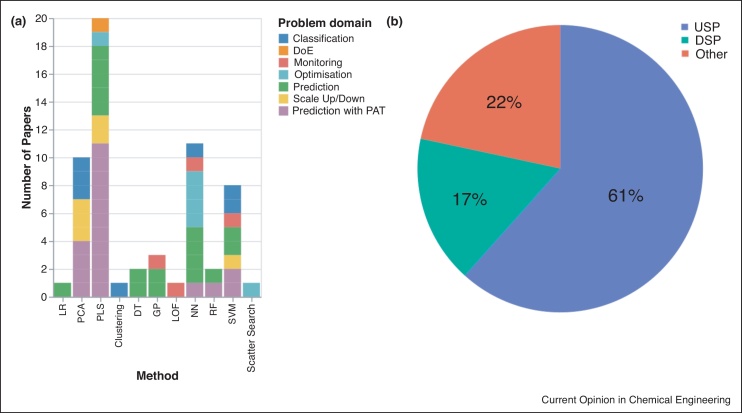
Applications of data analytics in the most cited journal articles by **(a)** problem domain per algorithm type and **(b)** process stage in terms of upstream processing (USP), downstream processing (DSP) or other.

Figure 4 shows the breakdown of each application area by problem domain and process stage. More specifically, Figure 4a shows the frequency of each problem domain per algorithm type for the 60 most cited journal articles from the period of 2015–2020 and highlights PLS was the most widely implemented and diverse algorithm in the sector. This was utilised in 33% of journal articles within five different problem domains. This is most likely due to its proven success within the sector over the last 30–40 years in analysing noisy data with strongly correlated variables [42], which are a common feature within biopharmaceutical data sets. Comparatively, NN were used in 18% of journal articles in Figure 4a and utilised within five different problem domains. For the purpose of clarification in this paper, ‘Prediction with PAT’ was defined as soft sensing using an external non-standard device such as Raman spectroscopy, while ‘Prediction’ referred to the use of a model to develop a soft sensor using currently available variables. The main application areas in terms of problem domain where data analytics have been applied are in ‘Prediction with PAT’ and ‘Prediction’ which, in total, account for 62% of the top 60 journal articles. With the increasing uptake of PAT across the sector, large amounts of data are being recorded, which can be better exploited using the available ML algorithms. Interestingly, the PAT applications in all 60 journal articles screened were focused on process monitoring with no demonstrations of control which, may indicate the utilisation of PAT within the biopharmaceutical sector is still in the early stages of deployment. A similar trend was identified by Armstrong *et al.* in bioprocess chromatography systems where there was a clear gap between the number of PAT applications used for monitoring in comparison to control. One of the challenges they discussed was related to lack of confidence in the application of these technologies from a regulatory approval perspective in comparison to the standard off-line quantification methods [43^•^]. While there are no control applications appearing in the analysis of the top cited literature, ML is still being utilised in the sector to optimise the performance of existing processes. NN have been able to identifying the optimal fermentation conditions of biopharmaceutical product [44], while also being utilised to increase the speed of parameter estimation in mechanistic modelling of chromatography runs [45].

Within the topmost cited 60 journal articles during this period, most techniques focused on upstream processing (USP) which accounts for 61% of the overall journal articles as seen in Figure 4b. This is due most likely to a greater number of variables recorded during USP operations in comparison to downstream processing (DSP). This emphasises an opportunity to further explore these techniques in DSP for process optimisation. It is clear that ML can provide additional insight into existing processes as seen in the volume of applications in Figure 4. The majority of these ML applications are generated within research and development environments and one of the remaining challenges will be simplifying the transfer of these models between different scales and processes. Craven *et al.* investigated and compared mechanistic and statistical model transferability across bioreactors of different scales and modes of operation in mammalian cell bioprocessing [46]. The authors found that for the prediction of viable cell density between batch, fed batch and continuous operations, the ML models prediction quality was lower compared to the mechanistic models. This was attributed to the ML model’s inability to incorporate feeding into its formulation, indicating that ML models may struggle to extrapolate from datasets which are widely different from the dataset it was trained and validated on. This research demonstrates that data analytics will continue to be an integral part of biopharmaceutical process development, but additional work is required to further exploit the benefits of these tools for process control and optimisation within commercial biomanufacturing.

## Conclusion

Over the last decade there has been a plethora of algorithms implemented for the analysis of highly diverse biopharmaceutical data sets. This work highlighted some interesting trends within the data set investigated. Between 2010 and 2020, PLS emerged as the most frequently applied algorithm within the sector, according to citation frequency. PLS represented 33% of such journal articles, rising to 60% when there were a large number of variables (>500). This accounted for almost 50% of the algorithm usage. This is most likely due to the high implementation of PLS for the analysis of PAT applications that contain large number of variables due to nature of the spectral data files. The second most cited algorithm was NN, with approximately 22% of journal articles published utilising this technique during this period. This may be due to the ability of this algorithm to capture complex relationships, which may yield more accurate predictions of non-linear variables such as viable cell densities or amino acid consumption rates. Within the journal articles evaluated, it was found that the majority of ML applications were focused on analysing data related to USP applications, accounting for 61% of the journal articles investigated. This is likely due to the large number of variables available for analysis compared to DSP or other application areas. There was no clear trend between the size of the data set analysed and the algorithm applied. This outcome demonstrates that the data set size, in terms of number of variables or experiments, is independent of the algorithm utilised. The appropriate algorithm should be based on the specific problem to be analysed. Therefore, the amount of data required for the development of useful models within the biopharmaceutical sector is most likely dependent on the complexity of both the data set and the problem to solve.

Based on the growing trend observed in the use of ML algorithms, it is clear that the sector will continue to explore and take advantage of insights and model predictions to optimise process development and manufacturing operations. Significant improvements are expected in current manufacturing operations with increased adoption of advanced data analytics, enabling soft sensor and PAT integration and hence more advanced control strategies. Furthermore, as the industry adopts the core principles of Industry 4.0, it will move towards the digitisation of all their recorded data within a queryable and structured centralised repository such as a data lake or data warehouse. This digital revolution will simplify data consolidation and accessibility, enabling ML algorithms to be applied to all data recorded from multiple sites across different scales and unit operations. This will help facilitate the ultimate goal of having a fully automated data-driven biopharmaceutical manufacturing facility of the future.

## Conflict of interest statement

Nothing declared.

## References and recommended reading

Papers of particular interest, published within the period of review, have been highlighted as:

• of special interest
•• of outstanding interest
